# Gentamicin Exposure and Sensorineural Hearing Loss in Preterm Infants

**DOI:** 10.1371/journal.pone.0158806

**Published:** 2016-07-08

**Authors:** Aline Fuchs, Lara Zimmermann, Myriam Bickle Graz, Jacques Cherpillod, Jean-François Tolsa, Thierry Buclin, Eric Giannoni

**Affiliations:** 1 Division of Clinical Pharmacology, Service of Biomedicine, Department of Laboratories, Lausanne University Hospital, Lausanne, Switzerland; 2 Clinic of Neonatology, Lausanne University Hospital, Lausanne, Switzerland; 3 Clinic of Otorhinolaryngology and Head and Neck Surgery, Lausanne University Hospital, Lausanne, Switzerland; Hôpital Robert Debré, FRANCE

## Abstract

**Objective:**

To evaluate the impact of gentamicin exposure on sensorineural hearing loss (SNHL) in very low birth weight (VLBW) infants.

**Methods:**

Exposure to gentamicin was determined in infants born between 1993 and 2010 at a gestational age < 32 weeks and/or with a birthweight < 1500 g, who presented with SNHL during the first 5 years of life. For each case, we selected two controls matched for gender, gestational age, birthweight, and year of birth.

**Results:**

We identified 25 infants affected by SNHL, leading to an incidence of SNHL of 1.58% in our population of VLBW infants. The proportion of infants treated with gentamicin was 76% in the study group and 70% in controls (*p* = 0.78). The total cumulated dose of gentamicin administered did not differ between the study group (median 10.2 mg/kg, Q1-Q3 1.6–13.2) and the control group (median 7.9 mg/kg, Q1-Q3 0–12.8, *p* = 0.47). The median duration of gentamicin treatment was 3 days both in the study group and the control group (*p* = 0.58). Maximum predicted trough serum levels of gentamicin, cumulative area under the curve and gentamicin clearance were not different between cases and controls.

**Conclusion:**

The impact of gentamicin on SNHL can be minimized with treatments of short duration, monitoring of blood levels and dose adjustment.

## Introduction

Preterm newborns are at risk of neurodevelopmental impairments such as cerebral palsy, cognitive, behavioral and sensory disabilities including hearing loss [1–3]. The incidence of sensorineural hearing loss (SNHL) is in the wide range of 0.4–5% among very low birthweight (VLBW) infants [2–8]. Therefore, screening for hearing loss is recommended during the neonatal period and during neurodevelopmental follow-up. A number of risk factors for SNHL have been described, including low gestational age and birthweight, intrauterine and postnatal infections, neonatal asphyxia, requirement for prolonged oxygen therapy and respiratory support, hyperbilirubinemia requiring exchange transfusion, hyponatremia, surgery during the neonatal period, congenital malformations, family history of hearing impairment, genetic abnormalities, and exposure to ototoxic medications such as diuretics and antibiotics [4, 9–23].

A large proportion of premature newborns are exposed to intravenous antibiotics. Indeed, invasive bacterial infection affects up to 25% of VLBW infants [24, 25]. The manifestations of infection are often unspecific and progression can be very rapid with a high risk of morbidity and mortality. Therefore, empirical antibiotic therapy is started at birth in the presence of risk factors for infection, and/or at the slightest suspicion of infection during the neonatal period. Gentamicin is one of the most commonly used antibiotics for empirical treatment of neonatal sepsis [26, 27]. Animal experiments and clinical studies on adults and children indicate that gentamicin can cause damage to the inner ear cells, resulting in irreversible SNHL. Gentamicin-related ototoxicity depends on the total dose administered, the duration of treatment, as well as serum concentrations [28]. Therapeutic drug monitoring (TDM) has thus been recommended during gentamicin treatment in order to achieve efficient serum concentrations of the drug and to minimize the risk of ototoxicity.

The impact of gentamicin treatment on the development of SNHL is controversial in newborns [29]. Early studies have found an association between aminoglycoside treatment and SNHL in newborn infants [12, 30]. A case-control study conducted at our institution between 1987 and 1991 identified the number of days of aminoglycoside treatment and the total cumulated dose of aminoglycosides as a risk factor for SNHL in premature infants [15]. However, since the early 1990s, a number of clinical studies have suggested that gentamicin administered in controlled therapeutic doses is not associated with ototoxicity in newborn infants [18, 21, 31–36]. Yet, controversies persist [17, 20, 23, 29, 37], and the dose, duration of treatment and circulating concentrations of gentamicin associated with the development of SNHL in VLBW infants have not been clearly determined.

Given the changes in the care of preterm neonates over the last twenty years, we decided to reassess the relationship between exposure to gentamicin and development of SNHL in premature newborns. We conducted a retrospective case-control study to determine the impact of gentamicin exposure and other potential risk factors on the development of SNHL in VLBW infants born between 1993 and 2010, and hospitalized at the University Hospital of Lausanne during the neonatal period.

## Patients and Methods

### Study design

This case-control study was approved by the local Human Research Ethics Committee (Commission cantonale d’éthique de la recherche sur l’être humain). Informed consent was not required due to the retrospective nature of the study. Patient information was anonymized and de-identified prior to analysis. Infants were included in the study group if they met all the following criteria: 1) date of birth between January 1, 1993 and December 31, 2010; 2) gestational age at birth < 32 weeks and/or birthweight < 1500 g; 3) hospitalization at the University Hospital of Lausanne during the neonatal period; 4) SNHL diagnosed during the first 5 years of life. For each case, we identified two controls matched for gender, gestational age, birthweight and year of birth.

### Audiologic assessment

Patients were identified through the database of the neonatal follow-up clinic of the University Hospital of Lausanne and the patient records of the pediatric otorhinolaryngologist of our institution. Testing for hearing impairment was performed before discharge in all infants born < 32 weeks of gestation and/or with a birthweight < 1500 g by a full diagnostic auditory brainstem response (ABR) until 2007, and by otoacoustic emissions from then on [38, 39]. Full diagnostic ABR testing was still performed after 2007 in cases of asphyxia, hyperbilirubinemia requiring exchange transfusion, and in the presence of major brain lesions or malformations associated with hearing loss. Patients with abnormal tests were referred to the pediatric otorhinolaryngologist for a comprehensive audiological evaluation including a full diagnostic ABR. All patients were assessed again at an age of 18 months, 3.5 years and 5 years by the Stycar hearing test or the whispered voice test [40]. Children with abnormal Stycar or whispered voice tests were referred to the pediatric otorhinolaryngologist for a comprehensive audiological evaluation by usual conditioned reaction audiometry or play audiometry, and a full diagnostic ABR.

### Gentamicin and aminoglycosides dosing regimens and therapeutic drug monitoring

Infants treated with gentamicin received an initial dose of 2.5–3 mg/kg. TDM was performed by measuring peak and trough gentamicin blood levels 1 and 12 hours after administration of the initial dose. Recommendations for gentamicin dose and interval were provided by clinical pharmacologists, based on a two-points linear regression [41], in order to achieve peak and trough gentamicin levels of 6–8 and 1–1.5 mg/L. Gentamicin peak and trough levels were controlled on the 4^th^ dose in infants treated for > 5 days or in the presence of renal failure and/or concomitant treatment with indomethacin, ibuprofen or furosemide. Infants treated with amikacin and tobramycin received an initial dose of 7.5–10 mg/kg and 2.5 mg/kg, and TDM was performed similarly to gentamicin.

### Risk factors and data collection

The primary causal attribute studied consisted in several descriptors of gentamicin treatment. The cumulated dose of gentamicin (mg/kg) was calculated by dividing the total dose administered to the patient by the patient’s body weight during gentamicin treatment. The duration of gentamicin treatment, as well as maximum measured peak and 12-hour plasma concentrations were recorded. Treatments with other aminoglycosides were also recorded. Gentamicin individual pharmacokinetic (PK) parameters were retrieved by maximum a posteriori Bayesian estimation [42], and were used to calculate the maximum predicted trough concentration during gentamicin treatment, the total cumulative area under the gentamicin concentration curve (AUC), and the systemic clearance of gentamicin.

Secondary study attributes included general clinical characteristics and conditions and other treatments identified as potential risk factors for SNHL in newborn infants [4, 9–15, 17–21]. Clinical data were retrieved from the database of the neonatal follow-up clinic and by medical chart review. We recorded data on medications, respiratory support and lung disease, hemodynamics, blood chemistry, infections, gastrointestinal complications, congenital malformations, chromosomal abnormalities and neurodevelopmental outcomes. The following neurodevelopmental impairments were recorded: 1) cognitive disability, defined by an intellectual quotient ≤ 2 standard deviations below the mean for the population; 2) cerebral palsy; 3) visual impairment not corrected by visual aids; 4) autistic spectrum disorder, defined as a behavioral disorder affecting communication and social skills.

### Statistical analyses

Baseline clinical characteristics were described by showing the median and the first and third quartiles (Q1–Q3) for continuous variables, and numbers and percentages for categorical variables. Group comparisons were performed using Mann–Whitney and Fisher’s Exact tests.

Associations between hypothesized risk factors and SNHL were evaluated using logistic regression analysis. Results were contrasted between the study group and the control group. Odd ratios and corresponding *p*-values were first assessed by univariate analyses. Associations that were evidently constructed were not tested (e.g. matching criteria). Then, candidate risk factors other than aminoglycosides that revealed a trend for an individual association with SNHL (*p* < 0.2) were entered into a stepwise multivariate analysis and backward eliminated until obtaining a parsimonious model that retained factors at least loosely associated with SNHL (*p* < 0.1). Eventually, the descriptors of gentamicin exposure were forced sequentially into the model to evaluate their own contribution. Statistical analyses were performed using the STATA software (version 13.1, StataCorp, Texas, USA).

## Results

A total of 1888 VLBW infants born between January 1, 1993 and December 31, 2010 were hospitalized at our institution. Their median gestational age was 29 5/7 weeks (Q1-Q3 27 4/7-31 0/7) and their median birthweight was 1195 g (Q1-Q3 910–1420). Two hundred and twenty six infants died during the neonatal period (12%) and 80 were lost to follow-up. Thus, 95% of the surviving patients were seen at the neonatal follow up clinic at least once, and 75% were evaluated up to 3 ½—6 years of age.

Among this population of 1582 VLBW infants, we identified a study group of 25 patients who developed SNHL during the first 5 years of life, leading to an incidence of SNHL of 1.58%. Demographic and perinatal characteristics were similar between cases and controls (Table 1). In the study group, one patient presented a congenital infection with cytomegalovirus, and one patient was affected by 18q- syndrome, a chromosomal abnormality associated with hearing impairment. No patient had an anomaly of ear or face, or a family history of hearing loss.

**Table 1:** Demographic and perinatal characteristics.

	study group (n = 25)	control group (n = 50)	*p* value
Female gender, n (%)	13 (52)	26 (52)	1.00
Gestational age, weeks [median (Q1-Q3)]	28 (26–31)	28 (26–31)	0.70
Birthweight, grams [median (Q1-Q3)]	780 (690–1100)	835 (660–1090)	0.91
Umbilical artery pH [median (Q1-Q3)]	7.27 (7.23–7.33)	7.27 (7.21–7.32)	0.93
Umbilical vein pH [median (Q1-Q3)]	7.31 (7.28–7.37)	7.34 (7.29–7.37)	0.53
1 min Apgar score [median (Q1-Q3)]	6 (2–7)	4 (3–6)	0.70
5 min Apgar score [median (Q1-Q3)]	8 (6–8)	7 (6–9)	0.83
10 min Apgar score [median (Q1-Q3)]	9 (7–9)	8 (7–9)	0.99
Cesarean section, n (%)	21 (84)	42 (84)	1.00
Antenatal steroids, n (%)	19 (76)	39 (78)	1.00
Preeclampsia, n (%)	5 (20)	7 (14)	0.52

Audiologic and neurodevelopmental outcomes of the study group are reported in Table 2. Twenty two patients had an abnormal ABR test during the neonatal period, 2 patients were diagnosed at 18 months, and 1 patient was diagnosed at 5 years. All patients with hearing loss were followed by an otorhinolaryngologist, and were treated with hearing aids, speech and language therapy. Besides SNHL, other severe neurodevelopmental impairments occurred in 11/25 (44%) of the patients from the study group, and 6/50 (12%) of the patients from the control group (*p* = 0.002).

**Table 2:** Audiologic status and neurodevelopmental outcome in the study group.

Patient No.	GA^a^ (weeks)	BW^b^ (grams)	ABR^c^ thresholdleft ear (dB)	ABR threshold right ear (dB)	Other severe impairments
1	28 5/7	1000	90	40	**-**
2	29	730	60	50	Cognitive disability^d^
3	28 4/7	1160	70	95	-
4	28 1/7	550	60	60	Cognitive disability, cerebral palsy
5	25 3/7	650	50	50	-
6	25 4/7	600	60	70	-
7	29 3/7	1000	70	70	-
8	31 1/7	780	100	30	-
9	25 5/7	580	0	50	Cognitive disability, cerebral palsy
10	25 2/7	770	70	60	Cognitive disability, visual impairment^e^
11	26	780	80	60	Cognitive disability, visual impairment
12	31	2030	40	40	Cognitive disability, cerebral palsy
13	24 2/7	690	0	60	-
14	26 6/7	610	70	40	-
15	32 5/7	1370	90	90	Cerebral palsy
16	27 5/7	900	60	60	-
17	26 6/7	910	90	90	-
18	31 4/7	1670	20	30	-
19	30 6/7	1600	40	70	Cognitive disability, cerebral palsy
20	32 1/7	860	50	40	Cognitive disability
21	31	1200	80	70	Cognitive disability, cerebral palsy
22	29 5/7	1100	60	60	-
23	24 5/7	720	90	90	Cognitive disability, cerebral palsy
24	25 4/7	700	60	90	-
25	27 1/7	475	50	90	-

^a^GA: gestational age

^b^BW: birthweight

^c^ABR: auditory brainsterm response

^d^Defined as an intellectual quotient ≤ 2 SD below the mean

^e^Not corrected by visual aids

The proportion of infants treated with gentamicin was 76% in cases vs 70% in controls (*p* = 0.78). The median duration of gentamicin treatment was 3 days (Q1-Q3 1–5) in cases and 3 days in controls (Q1-Q3 0–5, *p* = 0.58). The total cumulated dose of gentamicin did not differ between cases (median 10.2 mg/kg, Q1-Q3 1.6–13.2) and controls (median 7.9 mg/kg, Q1-Q3 0–12.8, *p* = 0.47, Table 3). Among infants treated with gentamicin, maximum observed peak and 12 h levels were similar between cases and controls. The univariate analysis of the derived gentamicin PK parameters did not show any significant difference between the two groups for cumulative AUC (*p* = 0.79), clearance (*p* = 0.78), and maximum predicted trough concentrations (*p* = 0.76). The mean cumulative AUC of gentamicin was 404 ± 217 mg/L/h in cases and 391 ± 162 mg/L/h in controls, with a 95% confidence interval (CI) for the difference between means of -84 to 109 [43].

**Table 3:** Gentamicin and other potentially ototoxic medications.

	study group (n = 25)	control group (n = 50)	*p* value
Gentamicin, n (%)	19 (76)	35 (70)	0.78
Duration of gentamicin treatment, days [median (Q1-Q3)]	3 (1–5)	3 (0–5)	0.58
Total cumulated dose of gentamicin, mg/kg [median (Q1-Q3)]	10.2 (1.6–13.2)	7.9 (0–12.8)	0.47
Maximum observed peak concentration of gentamicin, mg/L [median (Q1-Q3)]	5.7 (4.3–9.1)	5.6 (4.3–7.8)	0.67
Maximum observed 12 h concentration of gentamicin, mg/L [median (Q1-Q3)]	2.0 (1.7–2.4)	2.3 (1.8–2.6)	0.43
Maximum predicted trough concentration of gentamicin, mg/L [median (Q1-Q3)]	1.5 (1.3–1.8)	1.6 (1.3–1.8)	0.76
Cumulative AUC of gentamicin, mg/L/h [median (Q1-Q3)]	387 (223–539)	373 (288–478)	0.79
Clearance of gentamicin L/h/kg [median (Q1-Q3)]	0.037 (0.031–0.042)	0.036 (0.029–0.042)	0.78
Aminoglycoside, n (%)	20 (80)	39 (78)	1.00
Duration of aminoglycoside treatment, days [median (Q1-Q3)]	4 (2–7)	4 (2–7)	0.90
Median total cumulated dose of aminoglycosides, mg/kg (Q1-Q3)	17.5 (8.5–38.12)	12.7 (5.0–21.1)	0.15
Maximum observed peak concentration of aminoglycosides, mg/L [median (Q1-Q3)]	7.0 (5.0–11.6)	6.6 (4.4–8.8)	0.44
Maximum observed 12 h concentration of aminoglycosides, mg/L [median (Q1-Q3)]	2.0 (1.7–3.4)	2.4 (1.8–2.7)	0.41
Vancomycin treatment, n (%)	16 (64)	25 (50)	0.33
Furosemide treatment, n (%)	9 (36)	10 (20)	0.16
Neuromuscular blocking agents, n (%)	5 (20)	6 (12)	0.49

Four patients were treated with amikacin in the study group and 9 in the control group; one patient was treated with tobramycin in the study group, and one patient received netilmicin in the control group. The proportion of infants treated with any aminoglycoside was 80% among cases, and 78% among controls (*p* = 1.00). The total cumulated dose of aminoglycosides, the total duration of aminoglycoside treatment, and the maximum observed peak and 12 h levels of aminoglycosides were similar between cases and controls. The proportion of infants treated with furosemide, neuromuscular blocking agents and/or vancomycin was not different between cases and controls.

There was a higher incidence of pneumothorax in the study group than in the control group (20% vs 4%, *p* = 0.04, Table 4). Compared to controls, infants from the study group had a longer duration of mechanical ventilation and oxygen treatment, more frequently required catecholamine treatment for hypotension and as well as non-steroidal anti-inflammatory agents for patent ductus arteriosus. However, these differences did not reach statistical significance.

**Table 4:** General clinical data.

	study group (n = 25)	control group (n = 50)	*p* value
Invasive ventilation, n (%)	19 (76)	35 (70)	0.78
Duration of invasive ventilation, hours [median (Q1-Q3)]	144 (23–240)	75 (0–288)	0.47
Non-invasive ventilation, n (%)	21 (84)	45 (90)	0.47
Duration of non-invasive ventilation, hours [median (Q1-Q3)]	672 (288–1392)	696 (48–1240)	0.48
Duration of oxygen supplementation, hours [median (Q1-Q3)]	1008 (48–1704)	480 (24–1416)	0.21
Bronchopulmonary dysplasia^a^, n (%)	15 (60)	22 (44)	0.23
Pneumothorax, n (%)	5 (20)	2 (4)	0.04
Medically treated patent ductus arteriosus, n (%)	14 (56)	19 (38)	0.15
Surgically treated patent ductus arteriosus, n (%)	5 (20)	6 (12)	0.48
Hypotension treated with catecholamines, n (%)	7 (28)	8 (16)	0.23
Hyponatremia < 130 mmol/L, n (%)	5 (20)	12 (24)	0.78
Blood culture-proven sepsis, n (%)	8 (32)	8 (16)	0.14
Necrotizing enterocolitis^b^, n (%)	4 (17)	6 (12)	0.73
Gastrointestinal surgery, n (%)	1 (4)	1 (2)	1.00
Cerebral hemorrhage grade III or IV, n (%)	2 (8)	1 (2)	0.26
Periventricular leukomalacia, n (%)	3 (12)	2 (4)	0.33

^a^Defined as oxygen supplementation for > 28 days

^b^Bell stage ≥ 2

The multivariate analysis included risk factors unrelated to aminoglycosides with a *p*-value < 0.2. It produced a model which only retained pneumothorax and sepsis as factors overrepresented in patients with SNHL with odds ratios of 6.9 (95% CI 1.2–39.1, *p* = 0.03) and 2.8 (95% CI 0.9–8.9, *p* = 0.09) respectively. When parameters of exposure to gentamicin were serially entered in this reduced model one by one, none of them was significant (*p* > 0.5 for all). Altogether, the introduction of gentamicin treatment descriptors did not improve the model (*p* = 0.4, likelihood ratio test).

## Discussion

The impact of gentamicin on SNHL, once considered as major, is currently controversial in newborns. Here, we present a detailed analysis of gentamicin exposure in preterm infants that developed SNHL, and compare it with well-matched controls. Our results support the idea that gentamicin administration in therapeutically controlled doses is not associated with SNHL in VLBW infants.

The reported incidence of SNHL in VLBW infants varies widely between 0.4% and 5% in different studies [2–8]. At our institution, we found an incidence of SNHL of 1.58% in VLBW infants born between 1993 and 2010. Most of the patients were diagnosed during the neonatal period. In a study conducted in our neonatal intensive care unit in infants born < 35 weeks of gestation between 1987 and 1991, Borradori et al. found an incidence of SNHL of 1.46% [15]. The patients described by Borradori et al. were more mature (median gestational age 30 4/7) than the patients described in the present study (median gestational age 28 weeks). This difference is likely to reflect improvements in obstetric and neonatal intensive care during the past decades, resulting in the survival of more immature newborns. It is also noticeable that the exposure to gentamicin or other aminoglycosides reported by Borradori et al. was higher to the levels recorded in the present study, with average aminoglycoside treatment duration of 25 days, average cumulative gentamicin dose of 21.5 mg/kg, average trough concentrations of 3.0 mg/L, and frequent treatment rotation with tobramycin. This level of exposure, not unusual in the 1980s, was likely to be associated with ototoxic consequences.

In the present study, the majority of VLBW infants who developed SNHL were exposed to gentamicin, however at clearly lower doses, durations and concentrations than in the 1980s. Actually, there was no difference between cases and controls in the proportion of infants exposed to gentamicin, in the duration of gentamicin treatment, in the total cumulated dose, in maximum observed peak and 12 h gentamicin levels, and in individual estimates of gentamicin PK parameters (cumulative AUC, gentamicin clearance, maximum predicted trough level).

Overall, the rate of morbidities tended to be higher in patients affected by SNHL compared to controls. Pneumothorax is reported to occur in 2–10% of VLBW infants, depending on clinical practices for respiratory support [44, 45]. In the present study, the incidence of pneumothorax was 20% in the study group and 4% in controls, confirming the relationship between pneumothorax and SNHL that was previously identified at our institution [15]. Hypoxemia is a risk factor for hearing loss [21, 23]. We speculate that episodes of hypoxemia associated with pneumothoraces could have contributed to the development of SNHL. In contrast to previous studies, in our univariate analyses, we did not find associations between SNHL and several other known risk factors such as medication, respiratory and cardiovascular support, and surgical complications. However, there was a trend towards a higher use of furosemide and neuromuscular blocking agents, a higher rate of blood culture-proven sepsis, a longer duration of mechanical ventilation and oxygen therapy, and a higher incidence of patent ductus arteriosus in infants who developed SNHL. The proportion of infants affected by other severe neurodevelopmental disabilities in addition to their hearing impairment was higher in the study group compared to controls. This suggests that SNHL is multifactorial in origin, and part of a global neurodevelopmental impairment following complications of preterm birth.

The strength of the present study is the large number of VLBW infants included, along with a high rate of follow up, and a detailed analysis of gentamicin exposure. A limitation is the retrospective nature of the study. The relatively low number of patients affected by SNHL in our study is reassuring. On the other hand, this low number combined with a high variance in exposure to gentamicin lead to 95% CIs around the observed difference in gentamicin exposure that are consistent with as much as 25% higher exposure to gentamicin in infants who developed SNHL. Previous studies reporting on the absence of ototoxicity of gentamicin in newborns also suffer from methodological limitations including a small size, incomplete information on gentamicin exposure and absence of long term audiologic assessment [21, 33–36, 46, 47]. Hearing impairment may have resulted from the combined effect of prematurity, low birthweight, gentamicin treatment and other ototoxic medications such as furosemide and vancomycin, and genetic factors. Indeed, genetic variants can predispose to hearing loss. Mitochondrial DNA mutations have been linked to sensitivity to aminoglycosides and have also been associated with hearing loss in the absence of exposure to aminoglycosides [48]. Screening for genetic variants associated with hearing loss could identify patients at risk, but the opportunity for prevention may be limited as gentamicin is often prescribed on the first day of life for suspected early-onset sepsis [37, 49, 50]. We did not determine the prevalence of mutations predisposing for aminoglycoside toxicity. Vestibular toxicity was not investigated.

In conclusion, SNHL remains a serious complication of prematurity. Gentamicin is a potentially ototoxic medication. However, gentamicin-related ototoxicity can be minimized with relatively short treatments, close monitoring of blood levels, dosage adjustment, and ideally future genetic testing. A large prospective study with long term audiologic follow up is required to confirm our findings.

## Supporting Information

S1 File Table A. Demographic and perinatal characteristics in cases. Table B. Demographic and perinatal characteristics in controls. Table C. Gentamicin exposure in cases. Table D. Gentamicin exposure in controls. Table E. Exposure to potentially ototoxic medication in cases. Table F. Exposure to potentially ototoxic medication in controls. Table G. General clinical data in cases (1). Table H. General clinical data in controls (1). Table I. General clinical data in cases (2). Table J. General clinical data in controls (2).
(DOCX)
