# Supplementary material for: Gentamicin Exposure and Sensorineural Hearing Loss in Preterm Infants
Source: PLoS One. 2016 Jul 8;11(7):e0158806. doi: 10.1371/journal.pone.0158806 (PMC4938424; doi:10.1371/journal.pone.0158806)
Supplement: S1 File — Table A. Demographic and perinatal characteristics in cases. Table B. Demographic and perinatal characteristics in controls. Table C. Gentamicin exposure in cases. Table D. Gentamicin exposure in controls. Table E. Exposure to potentially ototoxic medication in cases. Table F. Exposure to potentially ototoxic medication in controls. Table G. General clinical data in cases (1). Table H. General clinical data in controls (1). Table I. General clinical data in cases (2). Table J. General clinical data in controls (2). (DOCX) [file pone.0158806.s001.docx]

**Table A. Demographic and perinatal characteristics in cases**

| **Number**  **(cases)** | **Gender** | **Year of birth** | **Gestational age (days)** | **Birth weight (grams)** | **Umbilical artery pH** | **Umbilical vein pH** | **1 min Apgar score** | **5 min Apgar score** | **10 min Apgar score** | **Cesarean section** | **Antenatal steroids** | **Pre-eclampsia** |
| --- | --- | --- | --- | --- | --- | --- | --- | --- | --- | --- | --- | --- |
| 1 | Male | 1993 | 201 | 1000 | 7,33 | 7,37 | 6 | 8 | 8 | Yes | Yes | No |
| 2 | Female | 1994 | 203 | 730 | 7,26 | 7,37 | 7 | 8 | 9 | Yes | No | No |
| 3 | Male | 1995 | 200 | 1160 | 7,33 | 7,37 | 7 | 8 | 8 | Yes | Yes | No |
| 4 | Female | 1996 | 197 | 550 | NA | 7,26 | 1 | 7 | 7 | Yes | Yes | No |
| 5 | Male | 1996 | 178 | 650 | NA | NA | 4 | 7 | 9 | No | No | No |
| 6 | Female | 1996 | 179 | 600 | 6,87 | 6,99 | 0 | 3 | 5 | No | Yes | No |
| 7 | Female | 1996 | 206 | 1000 | 7,24 | 7,29 | 2 | 2 | 3 | Yes | No | Yes |
| 8 | Male | 1997 | 218 | 780 | NA | NA | 3 | 7 | 9 | Yes | Yes | Yes |
| 9 | Female | 1997 | 180 | 580 | NA | 7,2 | 3 | 6 | 6 | No | No | No |
| 10 | Female | 1998 | 177 | 770 | 7,29 | 7,35 | 6 | 8 | 8 | Yes | Yes | No |
| 11 | Male | 1998 | 182 | 780 | 7,25 | 7,29 | 9 | 8 | 10 | Yes | Yes | No |
| 12 | Male | 1998 | 217 | 2030 | NA | NA | 1 | 2 | 5 | Yes | No | No |
| 13 | Female | 2000 | 170 | 690 | 7,22 | 7,3 | 6 | 8 | 9 | Yes | Yes | No |
| 14 | Female | 2001 | 188 | 610 | 7,33 | 7,34 | 7 | 9 | 10 | Yes | Yes | No |
| 15 | Male | 2002 | 229 | 1370 | 7,28 | 7,5 | 7 | 8 | 8 | Yes | Yes | Yes |
| 16 | Female | 2003 | 194 | 900 | 7,27 | 7,35 | 4 | 6 | 9 | Yes | Yes | No |
| 17 | Male | 2003 | 188 | 910 | 6,95 | 7,13 | 2 | 6 | 7 | Yes | Yes | Yes |
| 18 | Male | 2003 | 221 | 1670 | 7,34 | 7,4 | 8 | 9 | 10 | Yes | Yes | No |
| 19 | Male | 2005 | 216 | 1600 | NA | NA | 8 | 9 | 9 | Yes | Yes | No |
| 20 | Male | 2005 | 225 | 860 | 7,23 | 7,27 | 7 | 8 | 9 | Yes | Yes | No |
| 21 | Female | 2008 | 217 | 1200 | NA | NA | 0 | 0 | 0 | Yes | No | No |
| 22 | Female | 2009 | 208 | 1100 | 7,23 | 7,3 | 7 | 8 | 9 | Yes | Yes | No |
| 23 | Female | 2009 | 173 | 720 | 7,27 | 7,36 | 1 | 5 | 7 | Yes | Yes | No |
| 24 | Female | 2010 | 179 | 700 | NA | NA | 9 | 10 | 10 | No | Yes | No |
| 25 | Male | 2010 | 190 | 475 | 7,33 | 7,31 | 5 | 9 | 10 | Yes | Yes | Yes |

NA: not available

**Table B. Demographic and perinatal characteristics in controls**

| **Number**  **(controls)** | **Gender** | **Year of birth** | **Gestational age (days)** | **Birth weight (grams)** | **Umbilical artery pH** | **Umbilical vein pH** | **1 min Apgar score** | **5 min Apgar score** | **10 min Apgar score** | **Cesarean section** | **Antenatal steroids** | **Pre-eclampsia** |
| --- | --- | --- | --- | --- | --- | --- | --- | --- | --- | --- | --- | --- |
| 1 | Male | 1995 | 201 | 980 | 7,35 | 7,4 | 4 | 6 | 7 | Yes | Yes | Yes |
| 2 | Male | 1992 | 198 | 1040 | 7,31 | 7,37 | 6 | 7 | 8 | Yes | No | No |
| 3 | Female | 1993 | 203 | 750 | 7,22 | 7,27 | 7 | 8 | 9 | Yes | Yes | No |
| 4 | Female | 2011 | 201 | 750 | 7,27 | 7,38 | 9 | 10 | 10 | Yes | Yes | No |
| 5 | Male | 1995 | 197 | 1210 | 7,32 | 7,37 | 3 | 7 | 7 | Yes | Yes | No |
| 6 | Male | 1996 | 196 | 1110 | NA | NA | 5 | 6 | 6 | Yes | No | No |
| 7 | Female | 1995 | 196 | 560 | 7,29 | 7,47 | 6 | 9 | 9 | Yes | Yes | No |
| 8 | Female | 1997 | 196 | 600 | 7,21 | 7,27 | 1 | 3 | NA | Yes | Yes | Yes |
| 9 | Male | 1996 | 178 | 600 | 7,34 | 7,36 | 1 | 6 | 7 | Yes | Yes | No |
| 10 | Male | 1996 | 177 | 700 | 7,26 | 7,36 | 5 | 7 | 7 | No | No | No |
| 11 | Female | 1996 | 180 | 645 | 7,3 | 7,34 | 1 | 6 | 7 | Yes | Yes | No |
| 12 | Female | 2007 | 172 | 600 | 7,31 | 7,35 | 5 | 7 | 10 | Yes | Yes | No |
| 13 | Female | 1997 | 205 | 1035 | NA | NA | 4 | 8 | 8 | Yes | No | Yes |
| 14 | Female | 1994 | 203 | 1030 | 7,25 | 7,3 | 2 | NA | NA | Yes | Yes | No |
| 15 | Male | 1995 | 217 | 800 | 7,24 | 7,28 | 4 | 6 | 7 | Yes | No | No |
| 16 | Male | 1995 | 228 | 860 | 7,31 | NA | 5 | 7 | 8 | Yes | Yes | No |
| 17 | Female | 1998 | 176 | 560 | 7,16 | 7,28 | 2 | 6 | 8 | Yes | Yes | Yes |
| 18 | Female | 2001 | 183 | 560 | 7,2 | 7,2 | 1 | 5 | 5 | Yes | Yes | No |
| 19 | Female | 1997 | 175 | 730 | 7,34 | 7,4 | 3 | 7 | 7 | Yes | Yes | No |
| 20 | Female | 2000 | 175 | 760 | 7,25 | 7,35 | 3 | 6 | 9 | Yes | No | No |
| 21 | Male | 1998 | 186 | 750 | 7,13 | 7,2 | 2 | 7 | 7 | Yes | Yes | No |
| 22 | Male | 1997 | 186 | 810 | 7,32 | 7,36 | 4 | 6 | NA | No | Yes | No |
| 23 | Male | 1998 | 223 | 2060 | 7,18 | 7,29 | 8 | 9 | 9 | Yes | No | No |
| 24 | Male | 1992 | 223 | 1986 | 7,25 | 7,3 | 4 | 9 | 9 | Yes | Yes | No |
| 25 | Female | 2001 | 179 | 650 | 7,29 | 7,33 | 5 | 7 | 7 | Yes | Yes | No |
| 26 | Female | 2001 | 180 | 650 | 7,26 | 7,37 | 2 | 4 | 6 | Yes | Yes | No |
| 27 | Female | 2002 | 183 | 570 | 7,21 | 7,26 | 6 | 8 | 9 | Yes | Yes | Yes |
| 28 | Female | 2012 | 188 | 644 | 7,25 | 7,36 | 8 | 9 | 10 | Yes | Yes | No |
| 29 | Male | 2001 | 228 | 1330 | 7,11 | 7,14 | 6 | 9 | 9 | Yes | Yes | Yes |
| 30 | Male | 2004 | 226 | 1410 | 7,26 | 7,29 | 6 | 8 | 9 | Yes | Yes | No |
| 31 | Female | 2003 | 191 | 920 | 7,31 | 7,35 | 8 | 10 | 10 | No | Yes | No |
| 32 | Female | 2003 | 192 | 870 | 7,32 | 7,35 | 8 | 9 | 10 | Yes | Yes | No |
| 33 | Male | 2004 | 180 | 940 | 7,28 | 7,3 | 9 | 9 | 10 | Yes | Yes | No |
| 34 | Male | 2005 | 182 | 960 | NA | NA | 2 | 6 | 6 | No | No | No |
| 35 | Male | 2003 | 217 | 1670 | 7,17 | 7,22 | 3 | 9 | 9 | No | No | No |
| 36 | Male | 2001 | 218 | 1670 | 7,3 | 7,32 | 2 | 6 | 8 | Yes | Yes | No |
| 37 | Male | 2002 | 218 | 1580 | 7,26 | 7,34 | 9 | 10 | 10 | Yes | Yes | No |
| 38 | Male | 2008 | 215 | 1610 | 7,32 | 7,38 | 8 | 9 | 10 | Yes | Yes | No |
| 39 | Male | 1990 | 224 | 890 | 7,32 | 7,34 | 4 | 7 | 9 | Yes | Yes | No |
| 40 | Male | 2010 | 220 | 910 | 7,17 | 7,27 | 3 | 5 | 7 | Yes | Yes | No |
| 41 | Female | 2005 | 217 | 1220 | 6,8 | 6,8 | 3 | 7 | 8 | Yes | No | No |
| 42 | Female | 2010 | 216 | 1155 | 7,02 | NA | 7 | 9 | 9 | No | Yes | No |
| 43 | Female | 2008 | 206 | 1070 | 7,19 | 7,3 | 7 | 9 | 9 | Yes | Yes | No |
| 44 | Female | 2009 | 206 | 1100 | 7,29 | 7,38 | 5 | 8 | 9 | Yes | Yes | No |
| 45 | Female | 2010 | 174 | 700 | 7,33 | 7,37 | 2 | 4 | 7 | No | No | No |
| 46 | Female | 2008 | 175 | 710 | 7,32 | 7,38 | 3 | 8 | 9 | Yes | Yes | No |
| 47 | Female | 2009 | 179 | 720 | 7,35 | NA | 1 | 4 | 6 | Yes | Yes | No |
| 48 | Female | 2010 | 178 | 750 | 7,45 | 7,51 | 4 | 6 | 7 | No | Yes | No |
| 49 | Male | 2011 | 187 | 560 | 7,18 | 7,36 | 4 | 7 | 8 | Yes | Yes | Yes |
| 50 | Male | 1998 | 188 | 510 | 7,27 | 7,34 | 8 | 9 | 9 | Yes | Yes | No |

NA: not available

**Table C. Gentamicin exposure in cases**

| **Number**  **(cases)** | **Gentamicin treatment** | **Duration of gentamicin treatment (days)** | **Total cumulated dose of gentamicin(mg/kg)** | **Maximum observed peak concentration of gentamicin (mg/L)** | **Maximum observed 12 h concentration of gentamicin (mg/L)** | **Maximum predicted trough concentration of gentamicin (mg/L)** | **Cumulative AUC of gentamicin (mg/L/h)** | **Clearance of gentamicin (L/h/kg)** |
| --- | --- | --- | --- | --- | --- | --- | --- | --- |
| 1 | Yes | 2 | 6,7 | 3,7 | 2 | 1,04 | 155,03 | 0,043 |
| 2 | Yes | 4 | 10,2 | 4,8 | 2 | 1,56 | 386,46 | 0,036 |
| 3 | No | 0 | 0 | . | . | . | . | . |
| 4 | Yes | 3 | 5,4 | 4,3 | 7,1 | 1,44 | 445,16 | 0,022 |
| 5 | Yes | 10 | 11,2 | . | 2,3 | 1,45 | 504,81 | 0,034 |
| 6 | Yes | 4 | 7,5 | 5,5 | 3 | 1,48 | 464,2 | 0,027 |
| 7 | Yes | 3 | 10,5 | 3,8 | 1,7 | 2,63 | 377,69 | 0,028 |
| 8 | Yes | 1 | 1,6 | 9,2 | 1,7 | 1,03 | 45,379 | 0,045 |
| 9 | Yes | 8 | 10,2 | 7 | 1,7 | 2,62 | 577,05 | 0,030 |
| 10 | Yes | 4 | 11 | 9,2 | 2,5 | 2,56 | 339,92 | 0,042 |
| 11 | Yes | 3 | 16 | 6,6 | 2,4 | 2,01 | 587,25 | 0,035 |
| 12 | No | 0 | 0 | . | . | . | . |  |
| 13 | Yes | 7 | 13,1 | 9,4 | 1,9 | 1,78 | 538,45 | 0,035 |
| 14 | No | 0 | 0 | . | . | . | . | . |
| 15 | Yes | 7 | 33 | 14,6 | 1,4 | 1,51 | 648,97 | 0,037 |
| 16 | Yes | 5 | 13,7 | 9 | 1,8 | 1,63 | 404,17 | 0,038 |
| 17 | No | 0 | 0 | . | . | . | . | . |
| 18 | Yes | 3 | 16 | 4,2 | 1,8 | 1,27 | 211,41 | 0,045 |
| 19 | Yes | 5 | 24 | 5,1 | 1,7 | 1,63 | 368,22 | 0,041 |
| 20 | Yes | 5 | 12,2 | 5,7 | 2,2 | 1,25 | 346,92 | 0,041 |
| 21 | No | 0 | 0 | . | . | . | . | . |
| 22 | Yes | 3 | 13,2 | 3,6 | 1,6 | 1,12 | 223,22 | 0,054 |
| 23 | Yes | 13 | 21,7 | 8,7 | 3,7 | 1,75 | 966,03 | 0,031 |
| 24 | Yes | 2 | 2 | . | . | . | 76,276 | 0,037 |
| 25 | No | 0 | 0 | . | . | . | . | . |

**Table D. Gentamicin exposure in controls**

| **Number**  **(controls)** | **Gentamicin treatment** | **Duration of gentamicin treatment (days)** | **Total cumulated dose of gentamicin(mg/kg)** | **Maximum observed peak concentration of gentamicin (mg/L)** | **Maximum observed 12 h concentration of gentamicin (mg/L)** | **Maximum predicted trough concentration of gentamicin (mg/L)** | **Cumulative AUC of gentamicin (mg/L/h)** | **Clearance of gentamicin (L/h/kg)** |
| --- | --- | --- | --- | --- | --- | --- | --- | --- |
| 1 | Yes | 2 | 10,4 | 2,4 | 1,5 | 1,59 | 215,86 | 0,049 |
| 2 | No | 0 | 0 | . | . | . | . | . |
| 3 | No | 0 | 0 | . | . | . | . | . |
| 4 | Yes | 7 | 16,9 | 15,3 | 5,8 | 1,62 | 691,05 | 0,033 |
| 5 | Yes | 2 | 18 | 3,6 | 1,8 | 1,87 | 377,99 | 0,039 |
| 6 | Yes | 5 | 16,3 | 8,9 | 2,3 | 1,86 | 557 | 0,026 |
| 7 | Yes | 5 | 26,3 | 7,6 | 1,8 | 1,70 | 433,93 | 0,108 |
| 8 | Yes | 6 | 12 | 8,6 | 0,6 | 1,86 | 432,02 | 0,046 |
| 9 | Yes | 4 | 8,1 | 9,5 | 1,9 | 1,35 | 421,52 | 0,032 |
| 10 | Yes | 7 | 8,7 | 4,9 | 2,8 | 1,82 | 477,09 | 0,026 |
| 11 | Yes | 10 | 13,5 | 5,5 | 3,5 | 1,12 | 516,34 | 0,041 |
| 12 | Yes | 4 | 5,5 | 5 | 2,2 | 2,02 | 315,59 | 0,029 |
| 13 | No | 0 | 0 | . | . | . | . | . |
| 14 | Yes | 3 | 76,5 | 7,3 | 2,7 | 1,49 | 616,52 | 0,120 |
| 15 | No | 0 | 0 | . | . | . | . | . |
| 16 | No | 0 | 0 | . | . | . | . | . |
| 17 | Yes | 7 | 8,9 | 8,3 | 0,6 | 1,04 | 464,11 | 0,034 |
| 18 | Yes | 4 | 4 | 6 | 3,7 | 1,19 | 229,43 | 0,031 |
| 19 | Yes | 9 | 9 | 5,1 | 1,8 | 1,63 | 426,44 | 0,029 |
| 20 | Yes | 5 | 7,5 | 3,9 | 2,6 | 1,10 | 348,01 | 0,028 |
| 21 | Yes | 2 | 4,4 | 4,2 | 2,3 | 0,86 | 174,5 | 0,034 |
| 22 | No | 0 | 0 | . | . | . | . | . |
| 23 | No | 0 | 0 | . | . | . | . | . |
| 24 | No | 0 | 0 | . | . | . | . | . |
| 25 | No | 0 | 0 | . | . | . | . | . |
| 26 | Yes | 7 | 7,6 | 7 | 0,9 | 1,17 | 366,26 | 0,032 |
| 27 | Yes | 4 | 5,4 | 4,3 | 2,5 | 1,42 | 321,54 | 0,029 |
| 28 | Yes | 2 | 7,5 | 7,1 | 3,4 | 1,63 | 322,86 | 0,036 |
| 29 | No | 0 | 0 | . | . | . | . | . |
| 30 | No | 0 | 0 | . | . | . | . | . |
| 31 | Yes | 3 | 9,3 | 8,7 | 1,8 | 1,80 | 278,37 | 0,036 |
| 32 | Yes | 5 | 9,7 | 5,6 | 0,5 | 1,42 | 392,12 | 0,028 |
| 33 | Yes | 7 | 12,9 | 4,7 | 2,7 | 1,38 | 480,75 | 0,029 |
| 34 | Yes | 7 | 19,7 | 4,1 | 2 | 1,65 | 554,85 | 0,037 |
| 35 | Yes | 3 | 9,5 | 4,5 | 2,5 | 1,11 | 167,01 | 0,034 |
| 36 | Yes | 4 | 22,2 | 3,5 | 1,8 | 1,25 | 291,32 | 0,046 |
| 37 | Yes | 4 | 117,7 | 11 | 2,3 | 1,56 | 887,7 | 0,084 |
| 38 | Yes | 5 | 30 | 4,4 | 1,4 | 1,56 | 367,94 | 0,051 |
| 39 | No | 0 | 0 | . | . | . | . | . |
| 40 | No | 0 | 0 | . | . | . | . | . |
| 41 | Yes | 5 | 20 | 8,5 | 2,3 | 1,88 | 459,32 | 0,036 |
| 42 | Yes | 3 | 10,5 | 6,8 | 2,4 | 1,67 | 231,62 | 0,039 |
| 43 | No | 0 | 0 | . | . | . | . | . |
| 44 | Yes | 3 | 9,3 | 6,4 | 2,7 | 1,80 | 239,34 | 0,035 |
| 45 | Yes | 5 | 10 | 3,9 | 2 | 1,36 | 366,41 | 0,039 |
| 46 | Yes | 3 | 6,2 | 5,6 | 2,3 | 2,11 | 304,01 | 0,029 |
| 47 | Yes | 5 | 0 | . | . | . | . | . |
| 48 | Yes | 7 | 17,3 | 7,5 | 2,5 | 1,50 | 507,71 | 0,045 |
| 49 | No | 0 | 0 | . | . | . | . | . |
| 50 | Yes | 1 | 1,25 | 4,3 | 2,1 | . | 63,18 | 0,039 |

**Table E. Exposure to potentially ototoxic medication in cases**

| **Number**  **(cases)** | **Aminoglycoside treatment** | **Duration of aminoglycoside treatment (days)** | **Total cumulated dose of aminoglycosides (mg/kg)** | **Maximum observed peak concentration of aminoglycosides (mg/L)** | **Maximum observed 12 h concentration of aminoglycosides (mg/L)** | **Vancomycin treatment** | **Furosemide treatment** | **Neuromuscular blocking agents** |
| --- | --- | --- | --- | --- | --- | --- | --- | --- |
| 1 | Yes | 2 | 19,9 | 3,7 | 2 | Yes | Yes | Yes |
| 2 | Yes | 4 | 27,95 | 4,8 | 2 | No | No | Yes |
| 3 | No | 0 | 0 | . | . | Yes | No | Yes |
| 4 | Yes | 33 | 570,51 | 29,1 | 7,1 | Yes | Yes | No |
| 5 | Yes | 25 | 196,67 | 13,8 | 4,5 | No | Yes | Yes |
| 6 | Yes | 4 | 15,83 | 5,5 | 3 | Yes | No | No |
| 7 | Yes | 3 | 14,5 | 3,8 | 1,7 | Yes | No | No |
| 8 | Yes | 1 | 8,49 | 9,2 | 1,7 | Yes | No | Yes |
| 9 | Yes | 8 | 22,07 | 7 | 1,7 | Yes | No | No |
| 10 | Yes | 35 | 362,17 | 23,7 | 4,1 | Yes | Yes | No |
| 11 | Yes | 3 | 21,79 | 6,6 | 2,4 | Yes | Yes | No |
| 12 | No | 0 | 0 | . | . | No | No | No |
| 13 | Yes | 7 | 38,12 | 9,4 | 1,9 | Yes | Yes | No |
| 14 | No | 0 | 0 | . | . | Yes | No | No |
| 15 | Yes | 7 | 39,34 | 14,6 | 1,4 | No | No | No |
| 16 | Yes | 5 | 27 | 9 | 1,8 | No | No | No |
| 17 | Yes | 5 | 68,86 | 25,8 | 3,9 | Yes | Yes | No |
| 18 | Yes | 3 | 9,58 | 4,2 | 1,8 | No | No | No |
| 19 | Yes | 5 | 17,5 | 5,1 | 1,7 | Yes | No | No |
| 20 | Yes | 5 | 14,19 | 5,7 | 2,2 | No | No | No |
| 21 | No | 0 | 0 | . | . | No | No | No |
| 22 | Yes | 3 | 16,73 | 3,6 | 1,6 | No | No | No |
| 23 | Yes | 13 | 40,14 | 8,7 | 3,7 | Yes | Yes | No |
| 24 | Yes | 2 | 2,86 | . | . | Yes | Yes | No |
| 25 | No | 0 | 0 | . | . | Yes | No | No |

**Table F. Exposure to potentially ototoxic medication in controls**

| **Number**  **(controls)** | **Aminoglycoside treatment** | **Duration of aminoglycoside treatment (days)** | **Total cumulated dose of aminoglycosides (mg/kg)** | **Maximum observed peak concentration of aminoglycosides (mg/L)** | **Maximum observed 12 h concentration of aminoglycosides (mg/L)** | **Vancomycin treatment** | **Furosemide treatment** | **Neuromuscular blocking agents** |
| --- | --- | --- | --- | --- | --- | --- | --- | --- |
| 1 | Yes | 2 | 10 | 2,4 | 1,5 | No | No | No |
| 2 | No | 0 | 0 | . | . | No | No | No |
| 3 | No | 0 | 0 | . | . | Yes | No | No |
| 4 | Yes | 7 | 20 | 15,3 | 5,8 | Yes | No | No |
| 5 | Yes | 2 | 6,2 | 3,6 | 1,8 | No | No | Yes |
| 6 | Yes | 5 | 14,68 | 8,9 | 2,3 | Yes | Yes | Yes |
| 7 | Yes | 5 | 20,13 | 7,6 | 1,8 | No | Yes | No |
| 8 | Yes | 6 | 20 | 8,6 | 0,6 | Yes | No | Yes |
| 9 | Yes | 23 | 214,52 | 18,1 | 3,5 | No | No | No |
| 10 | Yes | 7 | 12,43 | 4,9 | 2,8 | No | Yes | No |
| 11 | Yes | 10 | 78,14 | 8 | 3,5 | Yes | Yes | No |
| 12 | Yes | 4 | 12,5 | 5 | 2,2 | Yes | No | No |
| 13 | No | 0 | 0 | . | . | No | No | No |
| 14 | Yes | 3 | 8,25 | 7,3 | 2,7 | Yes | No | No |
| 15 | Yes | 21 | 273 | 16,5 | 4,5 | No | Yes | No |
| 16 | No | 0 | 0 | . | . | No | No | Yes |
| 17 | Yes | 13 | 61,06 | 12,3 | 1,8 | Yes | Yes | No |
| 18 | Yes | 15 | 108,93 | 51,9 | 5,1 | Yes | No | No |
| 19 | Yes | 9 | 24,44 | 5,1 | 1,8 | No | No | Yes |
| 20 | Yes | 5 | 13,55 | 3,9 | 2,6 | Yes | No | No |
| 21 | Yes | 2 | 5,87 | 4,2 | 2,3 | Yes | No | No |
| 22 | Yes | 3 | 38,89 | 11,4 | 2,6 | No | No | Yes |
| 23 | No | 0 | 0 | . | . | No | No | No |
| 24 | No | 0 | 0 | . | . | No | No | No |
| 25 | Yes | 12 | . | . | . | Yes | No | No |
| 26 | Yes | 10 | 36,67 | 19,3 | 3,4 | Yes | No | No |
| 27 | Yes | 4 | 12,98 | 4,3 | 2,5 | Yes | No | No |
| 28 | Yes | 2 | 7,76 | 7,1 | 3,4 | Yes | Yes | No |
| 29 | No | 0 | 0 | . | . | No | No | No |
| 30 | No | 0 | 0 | . | . | No | No | No |
| 31 | Yes | 3 | 10 | 8,7 | 1,8 | No | Yes | No |
| 32 | Yes | 5 | 14,02 | 5,6 | 0,5 | No | No | No |
| 33 | Yes | 7 | 13,72 | 4,7 | 2,7 | Yes | Yes | No |
| 34 | Yes | 7 | 20,52 | 4,1 | 2 | No | No | No |
| 35 | Yes | 3 | 8,98 | 4,5 | 2,5 | No | No | No |
| 36 | Yes | 4 | 13,29 | 3,5 | 1,8 | No | No | No |
| 37 | Yes | 12 | 69,59 | 22,9 | 4 | Yes | No | No |
| 38 | Yes | 5 | 14,6 | 4,4 | 1,4 | No | No | No |
| 39 | No | 0 | 0 | . | . | Yes | No | No |
| 40 | Yes | 3 | 30 | . | . | No | No | No |
| 41 | Yes | 5 | 13,11 | 8,5 | 2,3 | No | No | No |
| 42 | Yes | 3 | 9,09 | 6,8 | 2,4 | No | No | No |
| 43 | No | 0 | 0 | . | . | No | No | No |
| 44 | Yes | 3 | 8,45 | 6,4 | 2,7 | Yes | No | No |
| 45 | Yes | 5 | 30 | 3,9 | 2 | Yes | No | No |
| 46 | Yes | 3 | 8,73 | 5,6 | 2,3 | Yes | No | No |
| 47 | Yes | 5 | . | . | . | Yes | No | No |
| 48 | Yes | 7 | 22,93 | 7,5 | 2,5 | Yes | No | No |
| 49 | No | 0 | 0 | . | . | Yes | Yes | No |
| 50 | Yes | 1 | 2,45 | 4,3 | 2,1 | Yes | No | No |

**Table G. General clinical data in cases (1)**

| **Number**  **(cases)** | **Invasive ventilation** | **Duration of invasion ventilation (hours)** | **Non invasive ventilation** | **Duration of non invasive ventilation (hours)** | **Duration of oxygen supplementation (hours)** | **Pneumothorax** | **Medically treated patent ductus arteriosus** | **Surgically treated patent ductus arteriosus** |
| --- | --- | --- | --- | --- | --- | --- | --- | --- |
| 1 | Yes | 216 | Yes | 528 | 456 | No | Yes | No |
| 2 | Yes | 120 | No | 0 | 504 | No | Yes | No |
| 3 | Yes | 220 | Yes | 408 | 1152 | Yes | Yes | No |
| 4 | Yes | 1608 | Yes | 288 | 5904 | No | Yes | Yes |
| 5 | Yes | 288 | Yes | 864 | 1176 | No | Yes | No |
| 6 | Yes | 744 | Yes | 1464 | 3072 | No | Yes | No |
| 7 | Yes | 240 | No | 0 | 480 | Yes | Yes | No |
| 8 | Yes | 23 | No | 0 | 6 | No | No | No |
| 9 | Yes | 288 | Yes | 312 | 1704 | No | No | No |
| 10 | Yes | 132 | Yes | 1416 | 1920 | No | No | Yes |
| 11 | Yes | 192 | Yes | 1392 | 1752 | Yes | Yes | No |
| 12 | No | 0 | No | 0 | 0 | No | No | No |
| 13 | Yes | 108 | Yes | 2208 | 1104 | Yes | Yes | Yes |
| 14 | No | 0 | Yes | 1200 | 1272 | No | Yes | No |
| 15 | No | 0 | Yes | 96 | 24 | No | No | No |
| 16 | No | 0 | Yes | 912 | 0,03 | No | No | No |
| 17 | Yes | 144 | Yes | 1680 | 1008 | No | Yes | No |
| 18 | No | 0 | Yes | 96 | 48 | Yes | No | No |
| 19 | Yes | 23 | Yes | 672 | 23 | No | No | No |
| 20 | No | 0 | Yes | 1392 | 960 | No | No | No |
| 21 | Yes | 192 | Yes | 480 | 1056 | No | No | No |
| 22 | Yes | 60 | Yes | 648 | 22 | No | No | No |
| 23 | Yes | 840 | Yes | 2832 | 3792 | No | Yes | Yes |
| 24 | Yes | 840 | Yes | 1728 | 3072 | No | Yes | Yes |
| 25 | Yes | 240 | Yes | 1320 | 1000 | No | Yes | No |

**Table H. General clinical data in controls (1)**

| **Number**  **(controls)** | **Invasive ventilation** | **Duration of invasion ventilation (hours)** | **Non invasive ventilation** | **Duration of non invasive ventilation (hours)** | **Duration of oxygen supplementation (hours)** | **Pneumothorax** | **Medically treated patent ductus arteriosus** | **Surgically treated patent ductus arteriosus** |
| --- | --- | --- | --- | --- | --- | --- | --- | --- |
| 1 | Yes | 77 | Yes | 40 | 117 | No | No | No |
| 2 | Yes | 18 | No | 0 | 18 | No | No | No |
| 3 | No | 0 | Yes | 17 | 360 | No | No | No |
| 4 | Yes | 480 | Yes | 1536 | 2712 | No | Yes | No |
| 5 | Yes | 72 | Yes | 72 | 182 | No | No | No |
| 6 | Yes | 264 | Yes | 120 | 528 | Yes | Yes | No |
| 7 | No | 0 | Yes | 48 | 1608 | No | No | No |
| 8 | Yes | 450 | Yes | 792 | 1372 | No | No | No |
| 9 | Yes | 168 | Yes | 48 | 264 | No | No | No |
| 10 | Yes | 144 | Yes | 672 | 1872 | No | No | No |
| 11 | Yes | 127 | Yes | 1181 | 1440 | No | No | No |
| 12 | Yes | 768 | Yes | 1296 | 1488 | No | Yes | No |
| 13 | Yes | 120 | Yes | 24 | 648 | No | No | No |
| 14 | Yes | 72 | No | 0 | 2976 | No | No | No |
| 15 | Yes | 41 | No | 0 | 43 | No | No | No |
| 16 | Yes | 20 | No | 0 | 24 | No | No | No |
| 17 | Yes | 1608 | Yes | 1 | . | No | Yes | No |
| 18 | Yes | 624 | Yes | 1464 | 1301 | No | No | No |
| 19 | Yes | 360 | Yes | 648 | 1584 | No | Yes | No |
| 20 | Yes | 0 | Yes | 1488 | 1248 | No | Yes | No |
| 21 | Yes | 140 | Yes | 800 | 888 | No | Yes | Yes |
| 22 | Yes | 120 | Yes | 800 | 900 | No | No | Yes |
| 23 | Yes | 0 | Yes | 72 | 0 | No | No | No |
| 24 | Yes | 42 | Yes | 2 | 96 | No | No | No |
| 25 | Yes | 192 | Yes | 1424 | 1704 | No | No | No |
| 26 | Yes | 1224 | Yes | 768 | 1104 | No | Yes | Yes |
| 27 | Yes | 408 | Yes | 2616 | 3000 | No | Yes | No |
| 28 | Yes | 86 | Yes | 1505 | 1416 | No | Yes | No |
| 29 | No | 0 | Yes | 36 | 0 | No | No | No |
| 30 | No | 0 | Yes | 8 | 0 | No | No | No |
| 31 | No | 0 | Yes | 720 | 24 | No | Yes | No |
| 32 | No | 0 | Yes | 504 | 96 | No | No | No |
| 33 | Yes | 288 | Yes | 1152 | 1032 | No | Yes | No |
| 34 | Yes | 288 | Yes | 1464 | 480 | No | Yes | No |
| 35 | No | 0 | Yes | 168 | 0 | No | No | No |
| 36 | No | 0 | Yes | 96 | 0 | No | No | No |
| 37 | No | 0 | Yes | 672 | 1 | No | No | No |
| 38 | No | 0 | Yes | 744 | 0 | No | No | No |
| 39 | Yes | 6 | No | 0 | 6 | No | No | No |
| 40 | No | 0 | Yes | 600 | 0 | No | No | No |
| 41 | Yes | 72 | Yes | 1260 | 96 | No | Yes | No |
| 42 | Yes | 24 | Yes | 312 | 79 | No | No | No |
| 43 | No | 0 | Yes | 864 | 0 | No | No | No |
| 44 | No | 0 | Yes | 1056 | 0 | No | No | No |
| 45 | Yes | 528 | Yes | 1272 | 1344 | No | Yes | No |
| 46 | Yes | 264 | Yes | 1656 | 1128 | No | Yes | Yes |
| 47 | Yes | 48 | Yes | 1152 | 24 | No | Yes | No |
| 48 | Yes | 672 | Yes | 1632 | 1776 | Yes | Yes | Yes |
| 49 | Yes | 628 | Yes | 1986 | 2688 | No | Yes | No |
| 50 | Yes | 336 | Yes | 888 | 1416 | No | No | No |

**Table I. General clinical data in cases (2)**

| **Number**  **(cases)** | **Hypotension treated with catecholamines** | **Hyponatremia < 130 mmol/L** | **Blood culture-proven sepsis** | **Necrotizing enterocolitis^a^** | **Gastrointestinal surgery** | **Cerebral hemorrhage grade III or IV** | **Periventricular leukomalacia** |
| --- | --- | --- | --- | --- | --- | --- | --- |
| 1 | No | No | No | No | No | No | No |
| 2 | No | No | No | No | No | No | No |
| 3 | No | No | No | No | No | No | No |
| 4 | Yes | Yes | Yes | Yes | Yes | No | No |
| 5 | No | No | No | Yes | No | No | No |
| 6 | No | No | Yes | No | No | No | No |
| 7 | No | No | Yes | No | No | No | No |
| 8 | No | No | Yes | No | No | No | No |
| 9 | No | No | No | No | No | Yes | No |
| 10 | No | No | Yes | Yes | No | No | No |
| 11 | No | No | No | No | No | No | No |
| 12 | No | No | No | No | No | No | No |
| 13 | Yes | Yes | No | No | No | No | No |
| 14 | Yes | Yes | No | No | No | No | No |
| 15 | No | No | No | No | No | No | No |
| 16 | No | No | Yes | No | No | No | No |
| 17 | Yes | No | No | Yes | No | No | No |
| 18 | No | No | No | No | No | No | No |
| 19 | No | No | No | No | No | No | Yes |
| 20 | No | No | No | No | No | No | No |
| 21 | Yes | No | No | No | No | No | No |
| 22 | Yes | No | No | No | No | No | No |
| 23 | Yes | Yes | Yes | No | No | No | Yes |
| 24 | No | Yes | Yes | No | No | Yes | No |
| 25 | No | No | No | No | No | No | Yes |

^a^Bell stage ≥ 2

**Table J. General clinical data in controls (2)**

| **Number**  **(controls)** | **Hypotension treated with catecholamines** | **Hyponatremia < 130 mmol/L** | **Blood culture-proven sepsis** | **Necrotizing enterocolitis^a^** | **Gastrointestinal surgery** | **Cerebral hemorrhage grade III or IV** | **Periventricular leukomalacia** |
| --- | --- | --- | --- | --- | --- | --- | --- |
| 1 | No | No | No | No | No | No | No |
| 2 | No | No | No | No | No | No | No |
| 3 | No | No | No | No | No | No | No |
| 4 | Yes | Yes | No | No | No | No | No |
| 5 | No | No | No | No | No | No | No |
| 6 | Yes | No | No | No | No | No | No |
| 7 | No | Yes | No | No | No | No | No |
| 8 | Yes | No | Yes | Yes | No | No | No |
| 9 | No | No | No | Yes | No | No | No |
| 10 | No | No | No | No | No | No | No |
| 11 | No | No | Yes | Yes | No | No | No |
| 12 | No | Yes | Yes | No | No | No | Yes |
| 13 | No | No | No | No | No | No | No |
| 14 | No | Yes | No | No | No | No | No |
| 15 | No | No | No | Yes | No | No | No |
| 16 | No | No | No | No | No | No | No |
| 17 | No | Yes | No | No | No | No | No |
| 18 | No | No | Yes | Yes | Yes | No | No |
| 19 | No | Yes | No | No | No | No | No |
| 20 | No | No | No | No | No | No | No |
| 21 | No | No | No | Yes | No | No | No |
| 22 | No | No | No | No | No | No | No |
| 23 | No | No | No | No | No | No | No |
| 24 | No | No | No | No | No | No | No |
| 25 | No | Yes | Yes | No | No | No | No |
| 26 | No | No | Yes | No | No | No | No |
| 27 | Yes | Yes | No | No | No | No | No |
| 28 | Yes | No | No | No | No | No | No |
| 29 | No | No | No | No | No | No | No |
| 30 | No | No | No | No | No | No | No |
| 31 | No | No | No | No | No | No | No |
| 32 | No | No | No | No | No | No | No |
| 33 | No | No | No | No | No | No | No |
| 34 | No | No | No | No | No | Yes | No |
| 35 | No | Yes | No | No | No | No | No |
| 36 | No | No | No | No | No | No | No |
| 37 | No | No | No | No | No | No | No |
| 38 | No | No | No | No | No | No | No |
| 39 | No | No | No | No | No | No | No |
| 40 | No | No | No | No | No | No | No |
| 41 | No | No | No | No | No | No | No |
| 42 | No | No | No | No | No | No | No |
| 43 | No | No | No | No | No | No | No |
| 44 | No | No | No | No | No | No | No |
| 45 | No | Yes | No | No | No | No | No |
| 46 | No | No | Yes | No | No | No | Yes |
| 47 | Yes | No | No | No | No | No | No |
| 48 | Yes | Yes | No | No | No | No | No |
| 49 | Yes | No | Yes | No | No | No | No |
| 50 | No | Yes | No | No | No | No | No |

^a^Bell stage ≥ 2
